# Differential Effects of sEH Inhibitors on the Proliferation and Migration of Vascular Smooth Muscle Cells

**DOI:** 10.3390/ijms18122683

**Published:** 2017-12-11

**Authors:** Hyo Seon Kim, Sang Kyum Kim, Keon Wook Kang

**Affiliations:** 1 College of Pharmacy and Research Institute of Pharmaceutical Sciences, Seoul National University, Seoul 08826, Korea; hyoseonkim@snu.ac.kr; 2 College of Pharmacy, Chungnam National University, Daejeon 34134, Korea; sangkim@cnu.ac.kr

**Keywords:** soluble epoxide hydrolase, epoxyeicosatrienoic acid, cyclooxygenase-2, vascular smooth muscle cell

## Abstract

Epoxyeicosatrienoic acid (EET) is a cardioprotective metabolite of arachidonic acid. It is known that soluble epoxide hydrolase (sEH) is involved in the metabolic degradation of EET. The abnormal proliferation and migration of vascular smooth muscle cells (VSMCs) play important roles in the pathogenesis of atherosclerosis and restenosis. Thus, the present study investigated the effects of the sEH inhibitor 12-(((tricyclo(3.3.1.13,7)dec-1-ylamino)carbonyl)amino)-dodecanoic acid (AUDA) on platelet-derived growth factor (PDGF)-induced proliferation and migration in rat VSMCs. AUDA significantly inhibited PDGF-induced rat VSMC proliferation, which coincided with Pin1 suppression and heme oxygenase-1 (HO-1) upregulation. However, exogenous 8,9-EET, 11,12-EET, and 14,15-EET treatments did not alter Pin1 or HO-1 levels and had little effect on the proliferation of rat VSMCs. On the other hand, AUDA enhanced the PDGF-stimulated cell migration of rat VSMCs. Furthermore, AUDA-induced activation of cyclooxygenase-2 (COX-2) and subsequent thromboxane A2 (TXA_2_) production were required for the enhanced migration. Additionally, EETs increased COX-2 expression but inhibited the migration of rat VSMCs. In conclusion, the present study showed that AUDA exerted differential effects on the proliferation and migration of PDGF-stimulated rat VSMCs and that these results may not depend on EET stabilization.

## 1. Introduction

Coronary heart disease is a leading cause of death in developed countries and accounts for one in seven deaths in the United States [1]. Restenosis, which is the re-narrowing of the vessel lumen after percutaneous coronary intervention (PCI), is mainly caused by neointimal hyperplasia [2,3]. In neointimal hyperplasia, endothelial injury activates platelets and induces the release of various growth factors and cytokines, including platelet-derived growth factor (PDGF). PDGF enhances the migration and proliferation of vascular smooth muscle cells (VSMCs) and fibroblasts in the media and adventitia of the vasculature, which subsequently contributes to the formation of neointima and reduces the size of the lumen [4,5,6]. Although there are conflicting perspectives regarding the origin of α-smooth muscle actin (α-SMA)-positive cells in the neointima, the proliferation and migration of VSMCs are considered to be the main factors contributing to neointimal hyperplasia [7,8,9].

Epoxyeicosatrienoic acid (EET), a bioactive product derived from the cytochrome P450-catalyzed pathway of arachidonic acid, has four regioisomers: 5,6-EET, 8,9-EET, 11,12-EET, and 14,15-EET. EET is hydrolyzed by soluble epoxide hydrolase (sEH) into dihydroxyeicosatrienoic acid (DHET), which is also active but readily degraded [10]. It is well-documented that EET exerts cardioprotective effects by preventing hypertension and reducing inflammation [11,12,13,14]. The EET receptor is yet to be characterized, but it is known that EET serves as a ligand for peroxisome proliferator-activated receptors (PPARs) [15]. Therefore, sEH inhibitors are being developed for the purpose of stabilizing endogenous EETs [16].

The present study aimed to determine the effects of sEH inhibitors on the proliferation and migration of VSMCs. Previous studies have shown that neointima formation induced by femoral cuff injury is diminished in hyperlipidemic sEH knockout mice [17], and urea-based sEH inhibitors, such as 1-cyclohexyl-3-dodecyl urea (CDU) and 12-(((tricyclo(3.3.1.13,7)dec-1-ylamino)carbonyl)amino)-dodecanoic acid (AUDA), inhibit the proliferation of PDGF-induced human aortic VSMCs [18,19]. Here, the sEH inhibitor AUDA suppressed rat VSMC proliferation but potentiated their migration, and that these effects might not be mediated through the stabilization of EETs. It was shown that AUDA inhibited PDGF-induced rat VSMC proliferation by decreasing peptidyl/prolyl isomerase pin1, while increasing heme oxygenase-1 (HO-1) expression. It was also demonstrated that AUDA induced the activation of cyclooxygenase-2 (COX-2) in PDGF-treated VSMCs, suggesting that surplus thromboxane A2 (TXA_2_) production might be related to enhanced VSMC migration.

## 2. Results

### 2.1. Effects of AUDA on VSMC Proliferation

**Figure 1:**
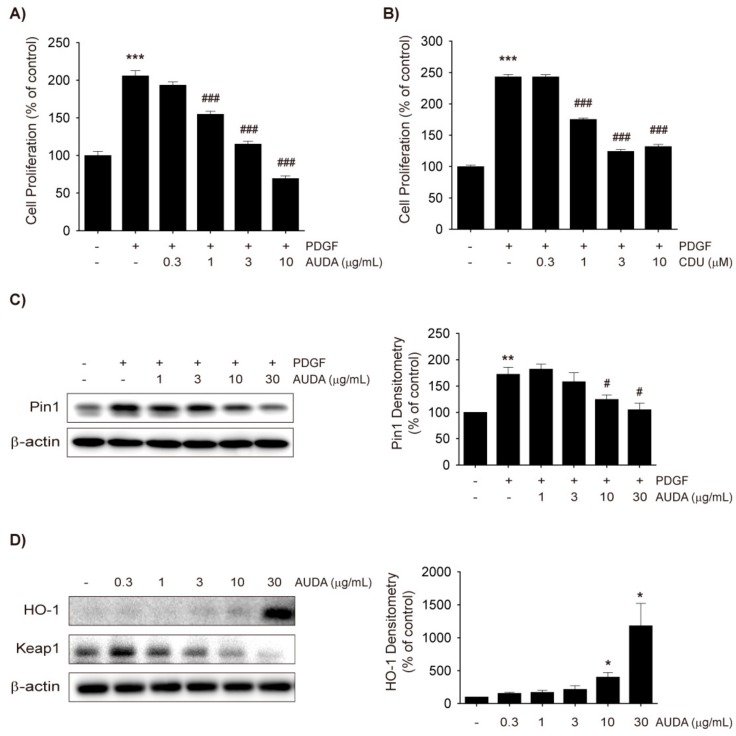

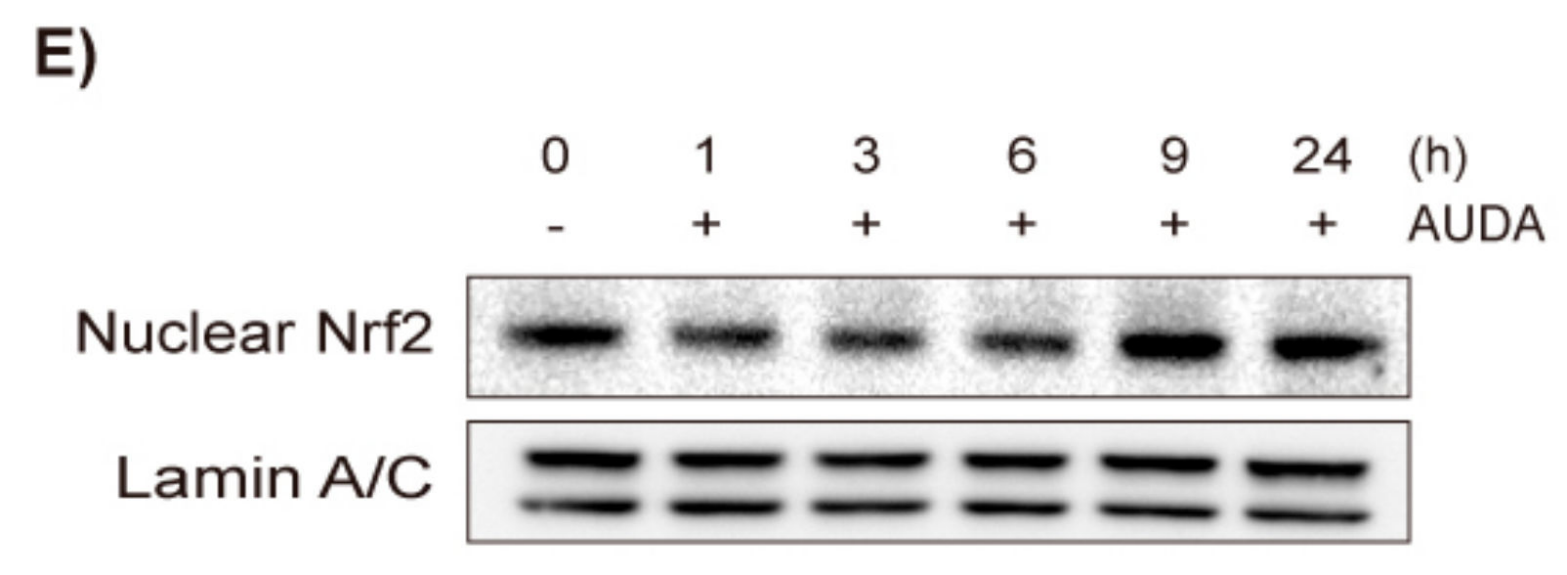
Soluble epoxide hydrolase (sEH) inhibitors inhibit platelet-derived growth factor (PDGF)-induced vascular smooth muscle cell (VSMC) proliferation by heme oxygenase-1 (HO-1) induction and Pin1 suppression. (**A**,**B**) Effects of 12-(((tricyclo(3.3.1.13,7)dec-1-ylamino)carbonyl)amino)-dodecanoic acid (AUDA). (**A**) and 1-cyclohexyl-3-dodecyl urea (CDU). (**B**) on VSMC proliferation. Rat VSMCs were treated with vehicle control, AUDA (0.3 to 10 μg/mL), or CDU (0.3 to 10 μM) 30 min prior to PDGF (30 ng/mL) exposure. VSMC proliferation was monitored 48 h after PDGF treatment by thiazolyl blue tetrazolium bromide (MTT) assay; (**C**) Effect of AUDA on Pin1 expression in VSMC. Vehicle control or AUDA (1 to 30 μg/mL) was treated 30 min prior to PDGF (30 ng/mL) exposure. Total cell lysates were obtained 24 h after PDGF treatment and subjected to Pin1 immunoblotting (*n* = 3); (**D**) Effects of AUDA on HO-1 and Kelch Like ECH Associated Protein 1 (Keap1) expression. VSMCs were incubated with vehicle control or AUDA (0.3 to 30 μg/mL) for 24 h and HO-1 and Keap1 expression was determined by immunoblottings (*n* = 3); (**E**) Effects of AUDA on nuclear level of nuclear factor erythroid 2-related factor-2 (Nrf2). VSMCs were incubated with vehicle control or 30 μg/mL AUDA for indicated time points and nuclear extracts were subjected to Nrf2 immunoblotting (*n* = 4). Statistical significance is indicated as * *p* < 0.05, ** *p* < 0.01, *** *p* < 0.001 vs. vehicle-treated control; ^#^ *p* < 0.05, ^###^ *p* < 0.001 vs. PDGF-treated group.

First, it was assessed whether the sEH inhibitors AUDA and CDU inhibit the PDGF-induced proliferation of rat VSMCs. Both AUDA and CDU dose-dependently suppressed the proliferation of rat VSMCs exposed to PDGF for 48 h (Figure 1A,B and Figure S1). Since CDU and AUDA are similar urea-based sEH inhibitors [19] and AUDA showed better efficacy, AUDA was mainly used in this study.

The peptidyl-prolyl isomerase Pin1 regulates proteins involved in cell cycle progression and apoptosis [20]. Our research group previously reported that the upregulation of Pin1 by PDGF inhibits the activation of nuclear factor erythroid 2-related factor-2 (Nrf2) and downregulates the level of HO-1 in VSMCs, which subsequently boosts VSMC proliferation [21]. Moreover, HO-1 expression in VSMCs has been shown to be inversely correlated with the formation of neointimal hyperplasia [22,23].

Thus, the present study investigated the effect of AUDA on Pin1 and Nrf2-mediated HO-1 expression in VSMCs. AUDA (1–30 μg/mL) dose-dependently inhibited the protein expression of Pin1 (Figure 1C) and increased HO-1 protein levels in PDGF-treated VSMCs at a dose of 3 μg/mL, although potent induction was seen only at 30 μg/mL (Figure 1D). To determine whether the increased HO-1 levels were dependent on the stability of the Kelch-like ECH-associated protein 1 (Keap1)-Nrf2 complex, Keap1 degradation, and Nrf2 nuclear translocation were examined. AUDA reduced Keap1 levels in a dose-dependent manner, and accordingly Nrf2 levels in the nucleus increased 9 h after compound treatment (Figure 1D,E). These data suggest that the sEH inhibitors dampen PDGF-induced VSMC proliferation, at least in part, by reducing Pin1 and enhancing HO-1 expression.

### 2.2. Effects of Exogenous EET on VSMC Proliferation

EETs induce HO-1 in the cardiovascular system; EET analogs increase HO-1 levels in human microvascular endothelial cells [24], mouse adipocytes [25], and the cardiac and adipose tissues of obese/diabetic mice [26]. In addition, the treatment of EET (1 μM) to human umbilical vein endothelial cells (HUVECs) has been shown to activate the Nrf2 pathway and increases HO-1 expression [27].

**Figure 2:**
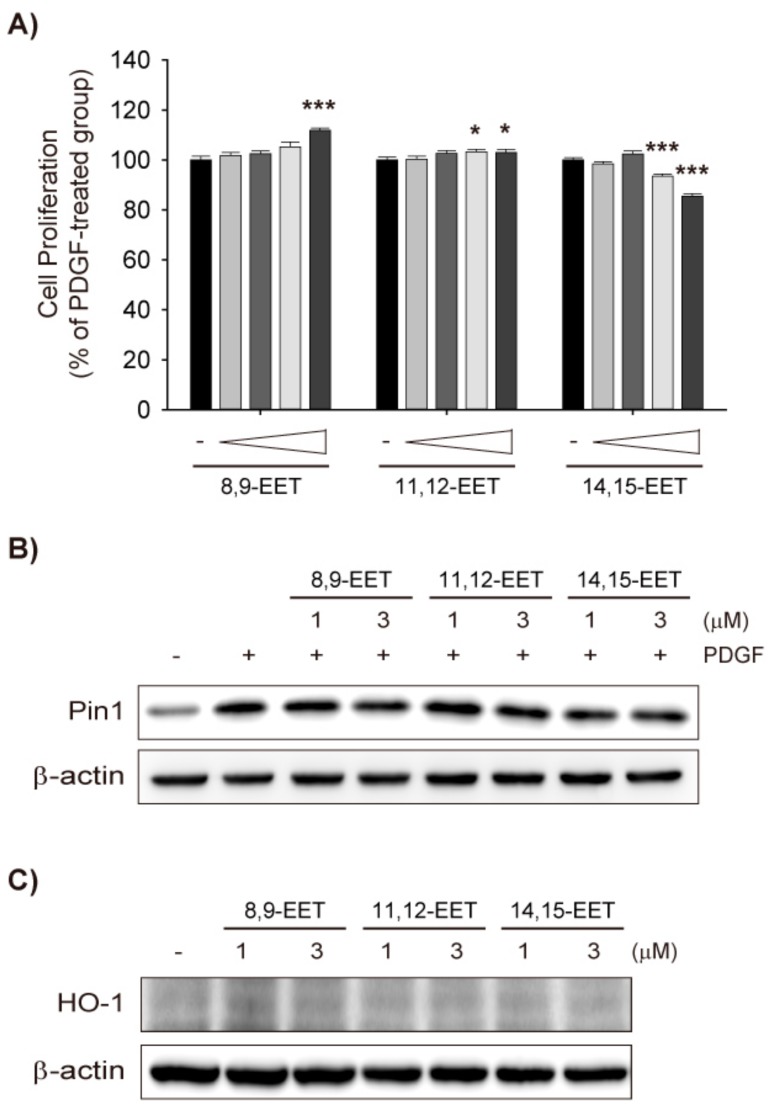
Exogenous epoxyeicosatrenoic acids (EETs) do not inhibit VSMC proliferation nor affect Pin1 and HO-1 expression. (**A**) Effects of EETs on VSMC proliferation. Rat VSMCs were preincubated with vehicle control, 8,9-EET, 11,12-EET, or 14,15-EET (0.1–3 μM) for 30 min and exposed to PDGF (30 ng/mL) for 48 h. VSMC proliferation was determined by MTT assay; (**B**) Effects of EETs on Pin1 expression. Rat VSMCs were pretreated with vehicle control, 8,9-EET, 11,12-EET, or 14,15-EET (1 or 3 μM) for 30 min and exposed to PDGF (30 ng/mL) for 24 h. Pin1 expression was determined by immunoblotting (*n* = 3); (**C**) Effect of EETs on HO-1 expression. HO-1 protein level was determined in VSMCs treated with vehicle control, 8,9-EET, 11,12-EET, or 14,15-EET (1 and 3 μM), for 24 h (*n* = 4). Statistical significance is indicated as * *p* < 0.05; *** *p* < 0.001 vs. PDGF-treated group.

Thus, the present study examined whether exogenous EET would inhibit VSMC proliferation by inducing HO-1 and inhibiting Pin1 as AUDA did. Because 11,12-EET and 14,15-EET are known to be the most abundant isomers in the vascular system, and 5,6-EET is barely detectable in human plasma [10,28], 8,9-EET, 11,12-EET, and 14,15-EET were used in this study. In rat VSMCs, 8,9-EET and 11,12-EET significantly enhanced PDGF-induced proliferation, while 14,15-EET significantly suppressed it (Figure 2A). However, the effective concentrations were considerably higher (3 μM) compared to human plasma concentrations (0.1–10 ng/mL) [28,29] and the degree of inhibition or enhancement was marginal (~15%). Furthermore, the three EET regioisomers did not reduce the Pin1 expression induced by PDGF (Figure 2B), nor did they increase HO-1 expression (Figure 2C). Since exogenous EETs do not mimic the effects of AUDA on VSMC proliferation, the anti-proliferative effects of sEH inhibitors may not be related to EET stabilization.

### 2.3. Differential Effects of AUDA and EET on VSMC Migration

**Figure 3:**
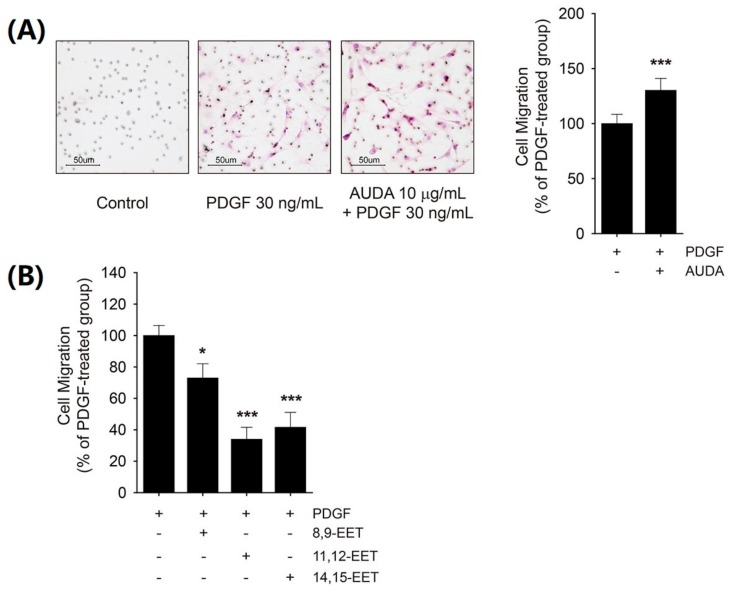
Differential effects of AUDA and EETs on PDGF-induced VSMC migration. (**A**) Effect of AUDA on PDGF-induced VSMC migration. Cell migration was determined via Boyden chamber assay in VSMC incubated with vehicle control or 10 μg/mL AUDA for 24 h. Culture media containing 30 ng/mL PDGF was added to the lower chamber as chemoattractant; (**B**) Effect of EETs on PDGF-induced VSMC migration. VSMCs were incubated with vehicle control, 8,9-EET, 11,12-EET, or 14,15-EET (1 μM, respectively) for 24 h. Cell migration was determined as described in (**A**). Statistical significance is indicated as * *p* < 0.05; *** *p* < 0.001 vs. PDGF-treated group.

Next, the effect of AUDA on VSMC migration was determined. A Boyden chamber migration assay revealed that PDGF increased VSMC migration and AUDA significantly enhanced PDGF-mediated VSMC migration (Figure 3A). However, AUDA alone did not induce VSMC migration (Figure 3A). On the contrary, all three EET regioisomers suppressed the migration of PDGF-stimulated VSMCs (Figure 3B). Taken together, it seems that the stimulatory effects of sEH inhibitors on VSMC migration are not dependent on the stabilization of EETs.

### 2.4. COX-2 Upregulation by AUDA and Its Involvement in Enhancing VSMC Migration

COX-2 induction and the subsequent overproduction of prostanoids in VSMCs, endothelial cells, and macrophages are involved in the pathogenesis of neointima formation in the vasculature [30]. Previous studies have shown that COX-2 knockout attenuated wire injury-mediated neointimal hyperplasia, and prostaglandin E2 (PGE_2_) receptor (EP) 3 deficiency prevented mouse VSMC migration [31]. It has been known that Angiotensin II and interleukin-1β induce COX-2 expression and VSMC migration, and inhibition of COX-2, EP, or the TXA_2_ receptor (TP) suppressed those effects [32], and PDGF also increases COX-2 expression and potentiates VSMC migration [33,34]. 

**Figure 4:**
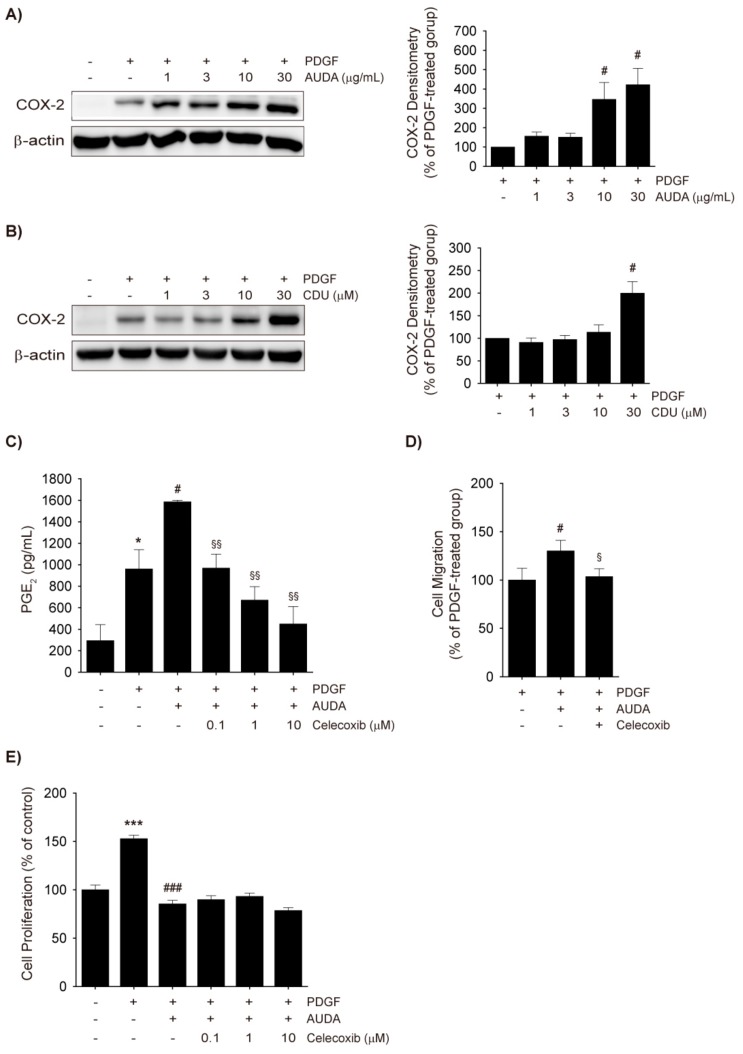
Cyclooxygenase-2 (COX-2) upregulation by sEH inhibitors and its role in VSMC migration. (**A**,**B**) COX-2 upregulation by sEH inhibitors. VSMCs were preincubated with vehicle control, AUDA ((**A**), 1–30 μg/mL), or CDU ((**B**), 1–30 μM) for 30 min, and then stimulated with 30 ng/mL PDGF for 8 h. The total cell lysates were subjected to COX-2 immunoblotting (*n* = 3); (**C**) PGE_2_ enzyme-linked immunosorbent assay (ELISA). Rat VSMCs were preincubated with vehicle control or AUDA (10 μg/mL) in the presence or absence of celecoxib (0.1–10 μM) for 30 min and then exposed to 30 ng/mL PDGF for 24 h. The secreted PGE_2_ levels in culture media were quantified by ELISA; (**D**) Effect of COX-2 inhibitor on VSMC migration. Cell migration was determined via Boyden chamber assay of VSMC incubated with vehicle control or 10 μg/mL AUDA with or without 1 μM celecoxib for 24 h. Culture media containing 30 ng/mL PDGF was added to the lower chamber as chemoattractant; (**E**) Effects of Celecoxib on VSMC proliferation. Rat VSMCs were preincubated with vehicle control or AUDA (10 μg/mL) in the presence or absence of celecoxib (0.1–10 μM) for 30 min and exposed to PDGF (30 ng/mL) for 48 h. VSMC proliferation was determined by MTT assay. Statistical significance is indicated as * *p* < 0.05; *** *p* < 0.001 vs. vehicle-treated control; ^#^ *p* < 0.05; ^###^ *p* < 0.001 vs. PDGF-treated group; ^§^ *p* < 0.05; ^§§^ *p* < 0.01 vs. PDGF + AUDA-treated group.

In the present study, PDGF increased COX-2 protein levels in rat VSMCs, and the sEH inhibitors AUDA and CDU dose-dependently upregulated COX-2 expression (Figure 4A,B). PGE_2_, a stable prostanoid, was quantified in the culture media to determine whether AUDA-induced increases in COX-2 expression would lead to increased COX-2 activity. Indeed, AUDA enhanced the production of PGE_2_ in PDGF-treated VSMCs, but this effect was dose-dependently diminished by co-treatment with the COX-2-selective inhibitor celecoxib (Figure 4C). In addition, the enhanced VSMC migration induced by AUDA was completely mitigated by low dose of celecoxib (1 μM; Figure 4D). The effects of COX-2 induction by sEH inhibitors on VSMC proliferation were also investigated. The co-incubation of VSMCs with celecoxib and AUDA caused neither an increase nor a decrease in PDGF-induced proliferation (Figure 4E). Thus, it seems plausible that the COX-2 upregulation by AUDA was associated with enhanced VSMC migration but not with the attenuated proliferation.

### 2.5. Role of TXA2 in sEH Inhibitor-Induced Enhancements in VSMC Migration

**Figure 5:**
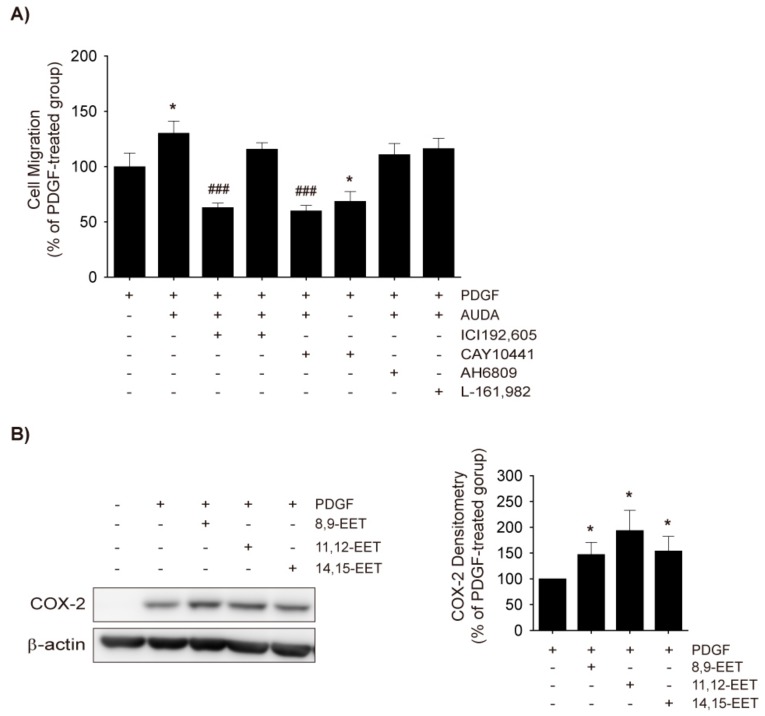
Role of thromboxna A2 (TXA_2_) in AUDA-stimulated VSMC migration. (**A**) Effects of prostanoid receptor antagonists on AUDA-stimulated VSMC migration. Rat VSMCs were treated with vehicle control, TXA_2_ receptor (TP) antagonist ICI192,605 (10 μM), PGI_2_ receptor (IP) antagonist CAY10441 (10 μM), EP1, EP2, EP3, and PGD_2_ receptor (DP)1 antagonist AH6809 (10 μM), or PGE_2_ receptor (EP) 4 antagonist L-161,982 (10 μM) in the presence or absence of AUDA (10 μg/mL) for 24 h. Cell migration was determined via Boyden chamber assay. Culture media containing 30 ng/mL PDGF was added to the lower chamber as chemoattractant; (**B**) Effects of EETs on COX-2 expression in VSMC. Rat VSMCs were preincubated with vehicle control, 8,9-EET, 11,12-EET, or 14,15-EET (1 μM) for 30 min and then exposed to 30 ng/mL PDGF for 8 h. COX-2 expression in total cell lysates was assessed by immunoblotting (*n* = 3). Statistical significance is indicated as * *p* < 0.05 vs. PDGF-treated group; ^###^ *p* < 0.001 vs. PDGF+AUDA-treated group.

Next, it was investigated whether COX-2-derived prostanoids would mediate the enhanced VSMC migration by AUDA. It has previously been shown that TXA_2_ and PGE_2_ induce VSMC migration [31,35]. Co-treatment of AUDA with various prostanoid receptor antagonists revealed that the TP antagonist ICI192,605 and the prostaglandin I2 (PGI_2_) receptor (IP) antagonist CAY10441, significantly prevented VSMC migration by AUDA while EP1, EP2, EP3, the prostaglandin D2 (PGD_2_) receptor (DP) antagonist AH6809, and the EP4 antagonist L-161,982 did not (Figure 5A). Because CAY10441 alone inhibited the PDGF-stimulated migration of VSMCs, its effects do not seem to coincide with AUDA co-treatment. Therefore, the enhancement of PDGF-induced VSMC migration by AUDA may be attributable to COX-2-mediated TXA_2_ production rather than EET stabilization.

It has been reported that 11,12-EET increases COX-2 levels and cell migration in HUVECs [36,37], while 14,15-EET inhibits PDGF-induced migration in human VSMC cell lines [38]. To assess the effects of EETs on COX-2 expression, rat VSMCs were treated with 8,9-EET, 11,12-EET, and 14,15-EET; all three EETs significantly enhanced PDGF-induced COX-2 expression in VSMCs (Figure 5B). Additionally, although the inhibition intensity was variable, 8,9-EET, 11,12-EET, and 14,15-EET significantly mitigated PDGF-induced VSMC migration (Figure 3B).

## 3. Discussion

The present study demonstrated that the sEH inhibitor AUDA suppressed rat VSMC proliferation via HO-1 upregulation and Pin1 downregulation. However, this study also showed that AUDA potentiated PDGF-stimulated VSMC migration via COX-2 induction and subsequent TXA_2_ overproduction. Unexpectedly, these differential effects were not reproduced by exogenous EETs. Thus, the effects of AUDA may or may not be related to sEH inhibition.

sEH inhibition led to decreased cell proliferation in VSMCs (Figure 1A,B) and, in fact, most urea-based sEH inhibitors suppress the proliferation of human VSMCs [18,19,38]. However, the effect of sEH inhibition on VSMC migration is yet to be confirmed. This study has demonstrated that both AUDA and CDU increased COX-2 expression in rat VSMCs (Figure 4A,B) and provided evidence that the potentiation of VSMC migration by sEH inhibitors was due to COX-2-catalyzed TXA_2_ formation (Figure 5A). On the other hand, Wang et al. [38] recently reported that the sEH inhibitor 1-(1-methanesulfonyl-piperidin-4-yl)-3-(4-trifluoromethoxy-phenyl)-urea (TUPS) inhibits PDGF-induced migration in a human aortic smooth muscle cell line. It remains unclear whether this discrepancy is due to differences in the chemical properties of the compounds or to differences between the human VSMC cell line and primary rat VSMCs.

The direct effects of EET on VSMC proliferation and migration are poorly understood. Davis et al. [18] reported that 10 μM of an EET mixture (8,9-EET, 11,12-EET, and 14,15-EET; 1:1:1) inhibits human VSMC proliferation, and that CDU suppresses VSMC proliferation via the downregulation of cyclin D1. Additionally, Wang et al. [38] showed that 100 nM 14,15-EET attenuates the migration of human VSMCs, while TUPS suppresses PDGF-induced VSMC switching from a contractile phenotype to a synthetic phenotype, in turn ameliorating neointimal hyperplasia. However, neither of these studies addressed the direct effects of EET on those mechanisms. In the present study, exogenous EETs did not suppress VSMC proliferation, nor did they affect Pin1 and HO-1 expression (Figure 2A–C). Although EETs either enhanced (8,9-EET and 11,12-EET) or suppressed (14,15-EET) VSMC proliferation at a concentration of 3 μM, the efficacy was marginal (~15%; Figure 2A). Furthermore, all three regioisomers of EET hindered the cell migration of PDGF-stimulated VSMCs (Figure 3B). Taken together, these results suggest that the aforementioned effects of sEH inhibitors might have been independent of EET stabilization.

PDGF-induced COX-2 upregulation is controlled by p38 mitogen-activated protein kinase (p38 MAPK) in VSMCs [39] and the activation of p38 MAPK in response to PDGF plays an important role in the migration of endothelial cells and VSMCs [33,40,41]. Moreover, increases in COX-2 activity cause Nrf2 activation and HO-1 induction, which are also regulated by p38 MAPK signaling, in the vasculature [42,43,44]. In this study, COX-2 expression occurred earlier than the nuclear translocation of Nrf2 and induction of HO-1 in AUDA-treated VSMCs (Figure 1C,D and Figure 4A). Because HO-1 expression was observed in the PDGF-free condition, it is difficult to determine whether the increased HO-1 levels were directly linked to the enhanced COX-2 expression. However, it seems probable that p38 MAPK signaling is associated with COX-2 upregulation and Nrf2 activation induced by sEH inhibition.

Even though COX-2 was induced by EETs in PDGF-treated VSMCs, EET did not enhance PDGF-stimulated VSMC migration, unlike AUDA (Figure 4A,B). Recent studies have shown that 5,6-EET, 8,9-EET, and 11,12-EET, but not 14,15-EET, can be metabolized by COX-2 to produce mitogenic and angiogenic metabolites (e.g., ct-8,9-epoxy-11-hydroxy-eicosatrienoic acid), which are then subjected to sEH metabolism [45,46]. Although their roles in the migration of VSMCs are yet to be elucidated, it seems possible that sEH inhibition by AUDA stabilizes these metabolites, as well as EET, thereby facilitating VSMC migration.

In conclusion, the sEH inhibitor AUDA suppressed proliferation while enhancing cell migration in PDGF-stimulated VSMCs. However, these effects of AUDA might not be related to EET stabilization because they were not mimicked by exogenous EET. Therefore, the development of sEH inhibitors for the purpose of alleviating neointimal hyperplasia should take these differential effects into consideration.

## 4. Materials and Methods

### 4.1. Antibodies and Reagents

12-(((tricyclo(3.3.1.13,7)dec-1-ylamino)carbonyl)amino)-dodecanoic acid (AUDA), 8,9-EET, 11,12-EET, 14,15-EET, CAY10441, L-161,982, and AH6809 were purchased from Cayman Chemical (Ann Arbor, MI, USA). 1-Cyclohexyl-3′-dodecylurea (CDU) and ICI-192,605 were obtained from Calbiochem (Darmstadt, Germany). PDGF was supplied by Peprotech (Rocky Hill, NJ, USA). Antibodies for Pin1, Keap1, Nrf2, COX-2 were purchased from Santa Cruz Biotechnology (Santa Cruz, CA, USA). HO-1 antibody and PGE2 enzyme-linked immunosorbent assay (ELISA) kit were obtained from Enzo Life Sciences (Ann Arbor, MI, USA), and Lamin A/C antibody was from Cell Signaling Technology (Beverly, MA, USA). Antibody for β–actin, thiazolyl blue tetrazolium bromide (MTT), elastase, Sulforhodamine B based in vitro toxicology assay kit, hematoxylin and eosin solution were supplied by Sigma-Aldrich (St. Louis, MO, USA). Collagenase was purchased from Worthington Biochemical Corporation (Lakewood, NJ, USA). HRP substrate kit was purchased from Millipore Corporation (Billerica, MA, USA). Transwell^®^ permeable supports were obtained from Corning Incorporated (Corning, NY, USA). DMSO was used as vehicle control for AUDA and CDU. Ethanol was used as vehicle control for EETs.

### 4.2. Isolation and Culture of VSMC

Rat aortas were isolated from 6–8-week-old Sprague Dawley rats. Connective tissues were removed from the aorta and adventitia were detached by incubation in collagenase. Remaining media were minced to pieces of 1–2 mm^2^ and incubated in collagenase and elastase for single cell suspension. VSMCs were cultured in Dulbecco’s Modified Eagle’s Medium (DMEM) with 10% fetal bovine serum (FBS), 100 U/mL penicillin, and 100 μg/mL streptomycin. VSMCs of 2–6 passages were used in the experiments.

### 4.3. Immunoblotting Analysis

Cells were harvested with phosphate buffered saline (PBS) and centrifuged at 3000× *g* for 5 min. Cell pellets were resuspended in cell lysis buffer—1 mM Tris pH 7.1, 100 mM NaCl, 1 mM EDTA, 10% glycerol, 0.5% Triton X-100, 0.5% Nonidet P-40 (NP-40), 1 mM dithiothreitol (DTT), and 0.5 mM phenylmethylsulfonyl fluoride (PMSF)—for 1 h on ice. The cell suspensions were centrifuged at 13,000× *g* for 15 min, and the resulting supernatants were used as total cell lysate. The protein samples were separated by sodium dodecyl sulfate-polyacrylamide gel electrophoresis (SDS-PAGE) and then transferred to nitrocellulose membrane. The membranes were blocked with 5% skim milk in PBS-Tween20 and incubated with primary antibodies as indicated. The membranes were washed and incubated with second antibodies conjugated with horseradish peroxidase (HRP). HRP substrates were added on the membrane and chemiluminescence was detected by LAS3000-mini (Fujifilm, Tokyo, Japan). The densitometry was measured using Image J software (NIH, Rockville, MD, USA).

### 4.4. Nuclear Fractionation

Cells were harvested with PBS and centrifuged at 3000× *g* for 5 min. Cell pellets were incubated in Buffer A—10 mM HEPES pH 7.9, 10 mM KCl, 0.1 mM EDTA, 1 mM DTT, 0.5 mM PMSF—for 10–12 min on ice. NP-40 with a 10% concentration was added to the mixture and vortexed and centrifuged at 16,000× *g* for 3 min. Resulting supernatants were used as cytosolic fractions. Remaining pellets were washed with Buffer A three times and with PBS once. The washed pellets were resuspended in Buffer B—20 mM HEPES pH 7.9, 0.4 M NaCl, 1 mM EDTA, 1 mM DTT, 1 mM PMSF—then vortexed in 4 °C for 10 min. The suspensions were centrifuged at 20,000× *g* for 15 min, and the supernatants were used as nuclear fractions.

### 4.5. MTT Proliferation Assay

VSMCs were seeded 20,000 cells per well on 96-well culture plate and cultured for 24 h, and the cells were serum-starved overnight. Cells were then treated as indicated for each experiment and incubated for 48 h. MTT solution was added to each well for a final concentration of 0.5 mg/mL and incubated for 2 h. Culture media was removed and the remaining formazan crystal was dissolved in dimethylsulfoxide (DMSO), and the absorbance was detected at 590 nm using a microtiter plate reader (Berthold Technology, Bad Wildbad, Germany).

### 4.6. Boyden Chamber Migration Assay

Transwell inserts were coated with type I collagen and left to dry. After they were completely dried, these inserts were transferred to a 24-well plate and VSMC suspensions in serum-free media with indicated reagents were seeded 1000 cells per well in the upper chamber of the inserts. PDGF was, when indicated, added to the lower chambers as a chemoattractant. Twenty-four hours later, the inserts were fixed in methanol and cells in the lower side of the inserts were stained with H&E. Cells were observed at 200× by phase contrast microscope and analyzed by number of cells on each field (*n* = 8).

### 4.7. ELISA

VSMCs were treated as indicated in the figure legends and incubated for 24 h, and supernatants were collected for PGE2 ELISA. PGE2 levels were measured according to the manufacturer’s instructions (Enzo, cat. No. ADI-900-001).

### 4.8. Sulforhodamine B Assay

VSMCs were seeded 20,000 cells per well on 96-well culture plate and cultured for 24 h, and the cells were serum-starved overnight. Cells were then treated as indicated for each experiment and incubated for 48 h. Sulforhodamine B staining was measured according to the manufacturer’s instructions (Sigma, cat. No. TOX6).

### 4.9. Statistical Analysis

SigmaPlot 12.0 software was used for data analysis. Statistical significance was determined via one-way ANOVA and *p* < 0.05 was considered significant. Data are shown in mean ± SEM.

## Supplementary Materials

Supplementary materials can be found at www.mdpi.com/1422-0067/18/12/2683/s1.

## Abbreviations

EET	epoxyeicosatrienoic acids
sEH	soluble epoxide hydrolase
VSMC	vascular smooth muscle cell
AUDA	12-(((tricyclo(3.3.1.13,7)dec-1-ylamino)carbonyl)amino)-dodecanoic acid
CDU	1-cyclohexyl-3-dodecyl urea
PDGF	platelet-derived growth factor
HO-1	heme oxygenase-1
COX-2	cyclooxygenase-2
Nrf2	nuclear factor erythroid 2-related factor-2
Keap1	Kelch like ECH associated protein 1
HUVEC	human umbilical vein endothelial cells
TXA_2_	thromboxane A2
TP	TXA_2_ receptor
PGE_2_	prostaglandin E2
EP	PGE_2_ receptor
PGI_2_	prostaglandin I2
IP	PGI_2_ receptor
PGD_2_	prostaglandin D2
DP	PGD_2_ receptor

## References

[1] Benjamin E.J., Blaha M.J., Chiuve S.E., Cushman M., Das S.R., Deo R., de Ferranti S.D., Floyd J., Fornage M., Gillespie C. (2017). Heart disease and stroke statistics-2017 update: A report from the american heart association. Circulation.

[2] Byrne R.A., Stone G.W., Ormiston J., Kastrati A. (2017). Coronary balloon angioplasty, stents, and scaffolds. Lancet.

[3] Buccheri D., Piraino D., Andolina G., Cortese B. (2016). Understanding and managing in-stent restenosis: A review of clinical data, from pathogenesis to treatment. J. Thorac. Dis..

[4] Elmore J.B., Mehanna E., Parikh S.A., Zidar D.A. (2016). Restenosis of the coronary arteries: Past, present, future directions. Interv. Cardiol. Clin..

[5] Raines E.W. (2004). PDGF and cardiovascular disease. Cytokine Growth Factor Rev..

[6] Chaabane C., Otsuka F., Virmani R., Bochaton-Piallat M.L. (2013). Biological responses in stented arteries. Cardiovasc. Res..

[7] Marx S.O., Totary-Jain H., Marks A.R. (2011). Vascular smooth muscle cell proliferation in restenosis. Circ. Cardiovasc. Interv..

[8] Yang P., Hong M.S., Fu C., Schmit B.M., Su Y., Berceli S.A., Jiang Z. (2016). Preexisting smooth muscle cells contribute to neointimal cell repopulation at an incidence varying widely among individual lesions. Surgery.

[9] He Y., Xu X., Zhu T., Tang M., Mei J., Si Y. (2017). Resident arterial cells and circulating bone marrow-derived cells both contribute to intimal hyperplasia in a rat allograft carotid transplantation model. Cell. Physiol. Biochem..

[10] Bellien J., Joannides R. (2013). Epoxyeicosatrienoic acid pathway in human health and diseases. J. Cardiovasc. Pharmacol..

[11] Oni-Orisan A., Alsaleh N., Lee C.R., Seubert J.M. (2014). Epoxyeicosatrienoic acids and cardioprotection: The road to translation. J. Mol. Cell. Cardiol..

[12] Thomson S.J., Askari A., Bishop-Bailey D. (2012). Anti-inflammatory effects of epoxyeicosatrienoic acids. Int. J. Vasc. Med..

[13] Imig J.D. (2015). Epoxyeicosatrienoic acids, hypertension, and kidney injury. Hypertension.

[14] Dong L., Zhou Y., Zhu Z.Q., Liu T., Duan J.X., Zhang J., Li P., Hammcok B.D., Guan C.X. (2017). Soluble epoxide hydrolase inhibitor suppresses the expression of triggering receptor expressed on myeloid cells-1 by inhibiting NF-κB activation in murine macrophage. Inflammation.

[15] Cowart L.A., Wei S., Hsu M.H., Johnson E.F., Krishna M.U., Falck J.R., Capdevila J.H. (2002). The cyp4a isoforms hydroxylate epoxyeicosatrienoic acids to form high affinity peroxisome proliferator-activated receptor ligands. J. Biol. Chem..

[16] Ingraham R.H., Gless R.D., Lo H.Y. (2011). Soluble epoxide hydrolase inhibitors and their potential for treatment of multiple pathologic conditions. Curr. Med. Chem..

[17] Revermann M., Schloss M., Barbosa-Sicard E., Mieth A., Liebner S., Morisseau C., Geisslinger G., Schermuly R.T., Fleming I., Hammock B.D. (2010). Soluble epoxide hydrolase deficiency attenuates neointima formation in the femoral cuff model of hyperlipidemic mice. Arterioscler. Thromb. Vasc. Biol..

[18] Davis B.B., Thompson D.A., Howard L.L., Morisseau C., Hammock B.D., Weiss R.H. (2002). Inhibitors of soluble epoxide hydrolase attenuate vascular smooth muscle cell proliferation. Proc. Natl. Acad. Sci. USA.

[19] Ng V.Y., Morisseau C., Falck J.R., Hammock B.D., Kroetz D.L. (2006). Inhibition of smooth muscle proliferation by urea-based alkanoic acids via peroxisome proliferator-activated receptor alpha-dependent repression of cyclin d1. Arterioscler. Thromb. Vasc. Biol..

[20] Litchfield D.W., Shilton B.H., Brandl C.J., Gyenis L. (2015). Pin1: Intimate involvement with the regulatory protein kinase networks in the global phosphorylation landscape. Biochim. Biophys. Acta.

[21] Kim S.E., Lee M.Y., Lim S.C., Hien T.T., Kim J.W., Ahn S.G., Yoon J.H., Kim S.K., Choi H.S., Kang K.W. (2010). Role of Pin1 in neointima formation: Down-regulation of Nrf2-dependent heme oxygenase-1 expression by Pin1. Free Radic. Biol. Med..

[22] Zhang M., Zhang B.H., Chen L., An W. (2002). Overexpression of heme oxygenase-1 protects smooth muscle cells against oxidative injury and inhibits cell proliferation. Cell Res..

[23] Yet S.F., Layne M.D., Liu X., Chen Y.H., Ith B., Sibinga N.E., Perrella M.A. (2003). Absence of heme oxygenase-1 exacerbates atherosclerotic lesion formation and vascular remodeling. FASEB J..

[24] Cao J., Tsenovoy P.L., Thompson E.A., Falck J.R., Touchon R., Sodhi K., Rezzani R., Shapiro J.I., Abraham N.G. (2015). Agonists of epoxyeicosatrienoic acids reduce infarct size and ameliorate cardiac dysfunction via activation of HO-1 and Wnt1 canonical pathway. Prostaglandins Other Lipid Mediat..

[25] Singh S.P., Schragenheim J., Cao J., Falck J.R., Abraham N.G., Bellner L. (2016). PGC-1 alpha regulates HO-1 expression, mitochondrial dynamics and biogenesis: Role of epoxyeicosatrienoic acid. Prostaglandins Other Lipid Mediat..

[26] Cao J., Singh S.P., McClung J.A., Joseph G., Vanella L., Barbagallo I., Jiang H., Falck J.R., Arad M., Shapiro J.I. (2017). EET intervention on Wnt1, NOV, and HO-1 signaling prevents obesity-induced cardiomyopathy in obese mice. Am. J. Physiol. Heart Circ. Physiol..

[27] Liu W.J., Wang T., Wang B., Liu X.T., He X.W., Liu Y.J., Li Z.X., Tan R., Zeng H.S. (2015). CYP2C8-derived epoxyeicosatrienoic acids decrease oxidative stress-induced endothelial apoptosis in development of atherosclerosis: Role of Nrf2 activation. J. Huazhong Univ. Sci. Technol. Med. Sci..

[28] Oni-Orisan A., Edin M.L., Lee J.A., Wells M.A., Christensen E.S., Vendrov K.C., Lih F.B., Tomer K.B., Bai X., Taylor J.M. (2016). Cytochrome P450-derived epoxyeicosatrienoic acids and coronary artery disease in humans: A targeted metabolomics study. J. Lipid Res..

[29] Long A., Zhong G., Li Q., Lin N., Zhan X., Lu S., Zhu Y., Jiang L., Tan L. (2015). Detection of 19 types of para-arachidonic acids in five types of plasma/serum by ultra performance liquid chromatography-tandem mass spectrometry. Int. J. Clin. Exp. Med..

[30] Weksler B.B. (2015). Prostanoids and nsaids in cardiovascular biology and disease. Curr. Atheroscler. Rep..

[31] Zhang J., Zou F., Tang J., Zhang Q., Gong Y., Wang Q., Shen Y., Xiong L., Breyer R.M., Lazarus M. (2013). Cyclooxygenase-2-derived prostaglandin E_2_ promotes injury-induced vascular neointimal hyperplasia through the E-prostanoid 3 receptor. Circ. Res..

[32] Aguado A., Rodriguez C., Martinez-Revelles S., Avendano M.S., Zhenyukh O., Orriols M., Martinez-Gonzalez J., Alonso M.J., Briones A.M., Dixon D.A. (2015). Hur mediates the synergistic effects of angiotensin II and IL-1beta on vascular COX-2 expression and cell migration. Br. J. Pharmacol..

[33] Yamaguchi H., Igarashi M., Hirata A., Sugae N., Tsuchiya H., Jimbu Y., Tominaga M., Kato T. (2004). Altered PDGF-BB-induced p38 MAP kinase activation in diabetic vascular smooth muscle cells: Roles of protein kinase C-delta. Arterioscler. Thromb. Vasc. Biol..

[34] Xu K., Kitchen C.M., Shu H.K., Murphy T.J. (2007). Platelet-derived growth factor-induced stabilization of cyclooxygenase 2 mRNA in rat smooth muscle cells requires the c-Src family of protein-tyrosine kinases. J. Biol. Chem..

[35] Feng X., Liu P., Zhou X., Li M.T., Li F.L., Wang Z., Meng Z., Sun Y.P., Yu Y., Xiong Y. (2016). Thromboxane A2 activates YAP/TAZ protein to induce vascular smooth muscle cell proliferation and migration. J. Biol. Chem..

[36] Michaelis U.R., Falck J.R., Schmidt R., Busse R., Fleming I. (2005). Cytochrome P4502C9-derived epoxyeicosatrienoic acids induce the expression of cyclooxygenase-2 in endothelial cells. Arterioscler. Thromb. Vasc. Biol..

[37] Michaelis U.R., Fisslthaler B., Barbosa-Sicard E., Falck J.R., Fleming I., Busse R. (2005). Cytochrome P450 epoxygenases 2C8 and 2C9 are implicated in hypoxia-induced endothelial cell migration and angiogenesis. J. Cell Sci..

[38] Wang Q., Huo L., He J., Ding W., Su H., Tian D., Welch C., Hammock B.D., Ai D., Zhu Y. (2015). Soluble epoxide hydrolase is involved in the development of atherosclerosis and arterial neointima formation by regulating smooth muscle cell migration. Am. J. Physiol. Heart Circ. Physiol..

[39] Yamaguchi H., Igarashi M., Hirata A., Tsuchiya H., Susa S., Tominaga M., Daimon M., Kato T. (2001). Characterization of platelet-derived growth factor-induced p38 mitogen-activated protein kinase activation in vascular smooth muscle cells. Eur. J. Clin. Investig..

[40] Matsumoto T., Yokote K., Tamura K., Takemoto M., Ueno H., Saito Y., Mori S. (1999). Platelet-derived growth factor activates p38 mitogen-activated protein kinase through a ras-dependent pathway that is important for actin reorganization and cell migration. J. Biol. Chem..

[41] Zhan Y., Kim S., Izumi Y., Izumiya Y., Nakao T., Miyazaki H., Iwao H. (2003). Role of jnk, p38, and erk in platelet-derived growth factor-induced vascular proliferation, migration, and gene expression. Arterioscler. Thromb. Vasc. Biol..

[42] Lim H.J., Lee K.S., Lee S., Park J.H., Choi H.E., Go S.H., Kwak H.J., Park H.Y. (2007). 15d-PGJ2 stimulates HO-1 expression through p38 map kinase and Nrf-2 pathway in rat vascular smooth muscle cells. Toxicol. Appl. Pharmacol..

[43] Park M.K., Kang Y.J., Lee H.S., Kim H.J., Seo H.G., Lee J.H., Chang K.C. (2008). The obligatory role of COX-2 expression for induction of HO-1 in ischemic preconditioned rat brain. Biochem. Biophys. Res. Commun..

[44] Luo C., Urgard E., Vooder T., Metspalu A. (2011). The role of COX-2 and Nrf2/ARE in anti-inflammation and antioxidative stress: Aging and anti-aging. Med. Hypotheses.

[45] Rand A.A., Barnych B., Morisseau C., Cajka T., Lee K.S.S., Panigrahy D., Hammock B.D. (2017). Cyclooxygenase-derived proangiogenic metabolites of epoxyeicosatrienoic acids. Proc. Natl. Acad. Sci. USA.

[46] Homma T., Zhang J.Y., Shimizu T., Prakash C., Blair I.A., Harris R.C. (1993). Cyclooxygenase-derived metabolites of 8,9-epoxyeicosatrienoic acid are potent mitogens for cultured rat glomerular mesangial cells. Biochem. Biophys. Res. Commun..

