# Supplementary material for: Differential Effects of sEH Inhibitors on the Proliferation and Migration of Vascular Smooth Muscle Cells
Source: Int J Mol Sci. 2017 Dec 11;18(12):2683. doi: 10.3390/ijms18122683 (PMC5751285; doi:10.3390/ijms18122683)
Supplement: Supplementary file 1 [file ijms-18-02683-s001.pdf]

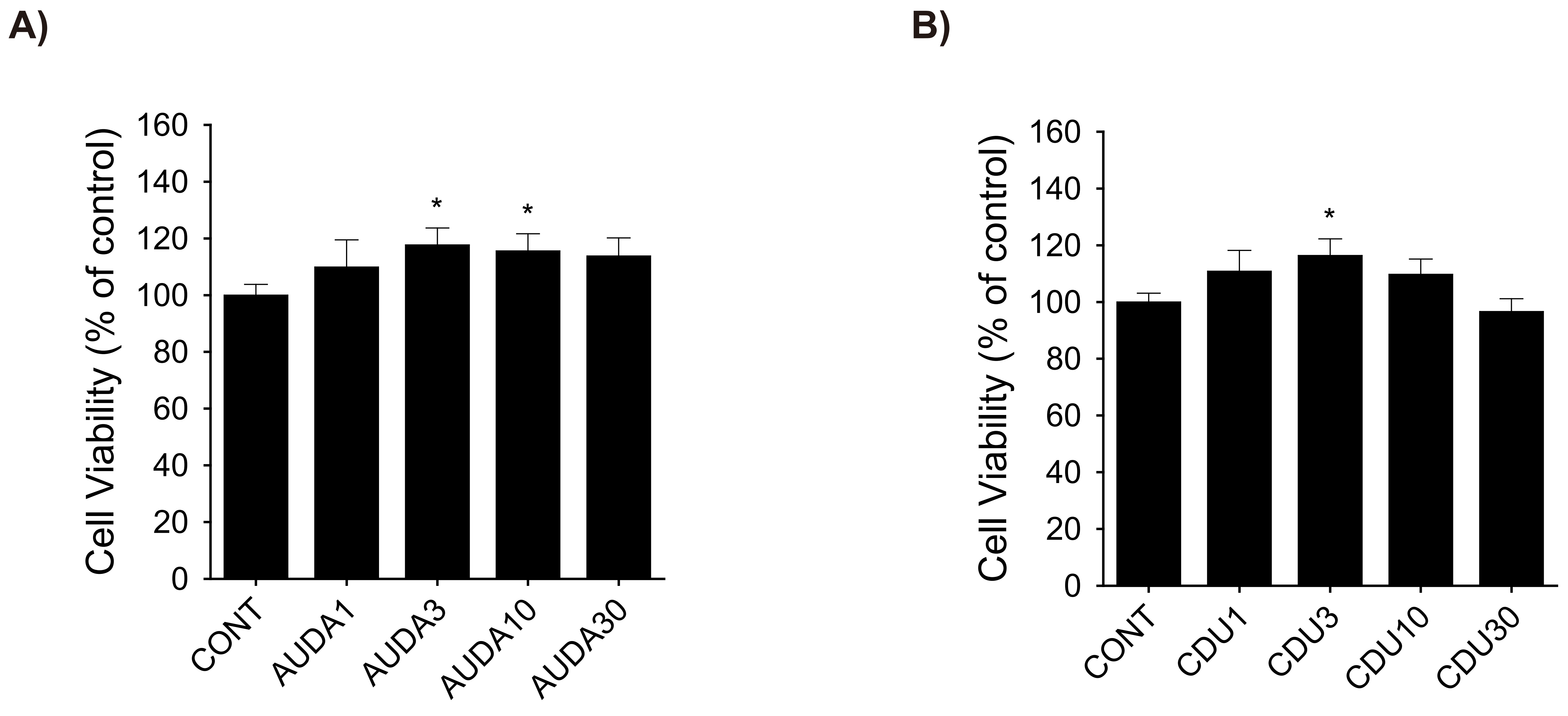

Figure S1. AUDA and CDU are not toxic to rat VSMCs. (**A, B**) Effects of AUDA (**A**) and CDU (**B**) on rat VSMC viability. Rat VSMC were treated with vehicle, AUDA (1 to 30 μg/mL) or CDU (1 to 30 μM) for 48 h. VSMC viability was measured by Sulforhodamine B assay. Statistical significance is indicated as *p<0.05 vs control.
